# Key Features in the Management of Pulmonary Carcinosarcoma

**DOI:** 10.1155/2016/2020146

**Published:** 2016-02-18

**Authors:** Nikolaos Panagiotopoulos, Davide Patrini, Benjamin Adams, Jonathan Pararajasingham, Rajeev Shukla, Elaine Borg, Martin Hayward, David Lawrence

**Affiliations:** ^1^Department of Cardiothoracic Surgery, University College London Hospitals (UCLH), 16-18 Westmoreland Street, London W1G 8PH, UK; ^2^Department of Pathology, University College London Hospitals (UCLH), 21 University Street, Rockefeller Building, London WC1E 6DE, UK

## Abstract

Pulmonary carcinosarcoma represents a category of extremely rare tumours accounting for 0.1% of all lung malignancies. It is defined as a poorly differentiated non-small-cell carcinoma that contains a component of sarcoma or sarcoma-like elements. These biphasic tumours typically have a poor prognosis due to late diagnosis and early metastases. Preoperative tissue diagnosis is usually difficult due to the heterogeneity of the tumour, with biopsies often just reflecting one element of the tumour. By means of a case illustration and review of the literature, we discuss the optimal management of patients with pulmonary carcinosarcoma.

## 1. Introduction

Pulmonary carcinosarcoma can be defined as a poorly differentiated non-small-cell carcinoma that contains a component of sarcoma or sarcoma-like elements. They are extremely rare tumours, accounting for only 0.1% of all lung malignancies [1]. It remains a point of contention as to whether carcinosarcoma truly exists as a distinct pathological entity. In actuality these tumours are histologically heterogeneous and therefore likely represent a continuum of epithelial and mesenchymal differentiation [1]. Preoperative tissue diagnosis is consequently difficult due to the heterogeneity of the tumour, with biopsy often just reflecting one element of the tumour. Similarly there are no clear findings on imaging which indicate a likely diagnosis. Given the associated or perceived worse prognosis when compared with other types of NSCLC, we look to try to answer what is the optimal management of patients with pulmonary carcinosarcoma.

## 2. Illustrative Case Presentation

We present the case of an 83-year-old lady who had been followed up over a three-year period with a small peripheral right upper lobe lesion. She underwent repeated surveillance imaging with PET CT. The mass was of low avidity and the density of the mass appeared to wax and wane over the years. The patient also had repeated bronchoscopies with endobronchial washings and biopsies, which were negative for malignancy. At her most recent interval scan however the mass had grown rapidly in size to over 3 cm.

Following discussion at the lung cancer MDT (Multidisciplinary Team) meeting, she was clinically staged as a T2b N0 M0 lung cancer. Her lung function tests were excellent with an FEV1 of 109% predicted and a TLCO of 83%. The patient was referred to our service and underwent successful surgical management with a right thoracotomy and upper lobectomy. She was treated for fast response atrial fibrillation in the postoperative period but otherwise the patient made an excellent recovery and was discharged on the eighth day following surgery.

This case illustrates the diagnostic uncertainty of managing patients with what at first appeared to be a benign solitary pulmonary nodule.

## 3. Histology Report

**Figure 1:**
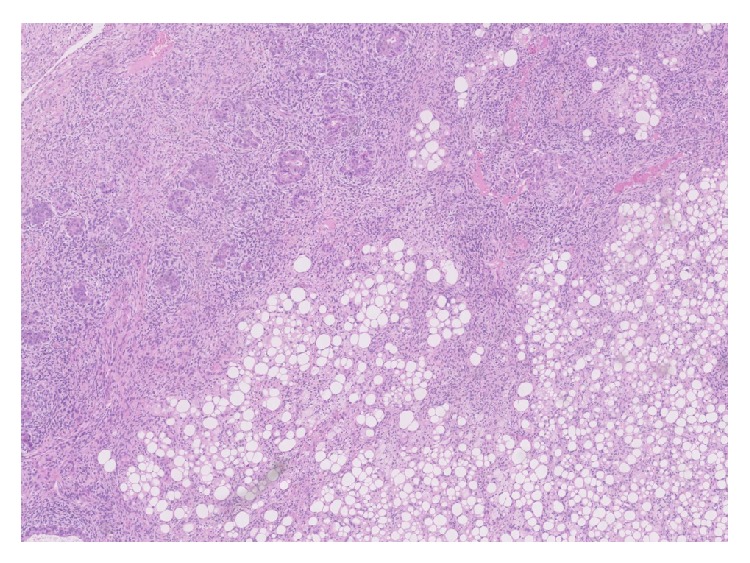
Lung parenchyma showing infiltration by biphasic malignant tumour which in areas shows lipoblastic elements (H&E ×5 objective).

**Figure 2:**
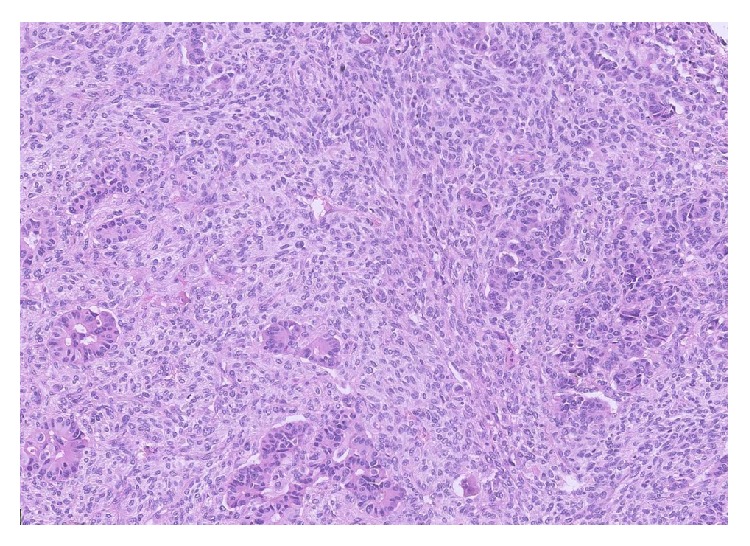
Malignant poorly formed epithelial glands with surrounding malignant spindle cell sarcoma (H&E ×10 objective).

**Figure 3:**
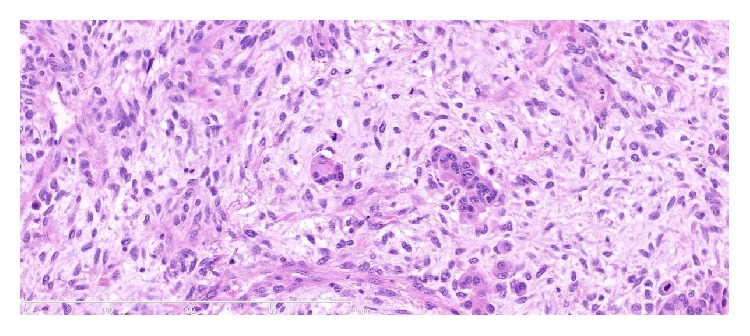
High power photomicrograph picture (Mag ×20) showing carcinoma with atypical glands and atypical stroma showing increased mitotic activity.

**Figure 4:**
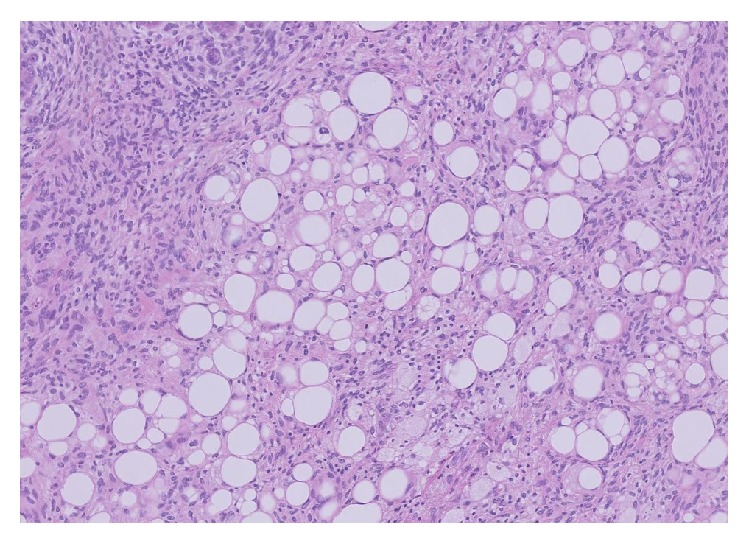
Lipoblastic elements seen admixed with the spindle cell component (H&E ×10 objective).

**Figure 5:**
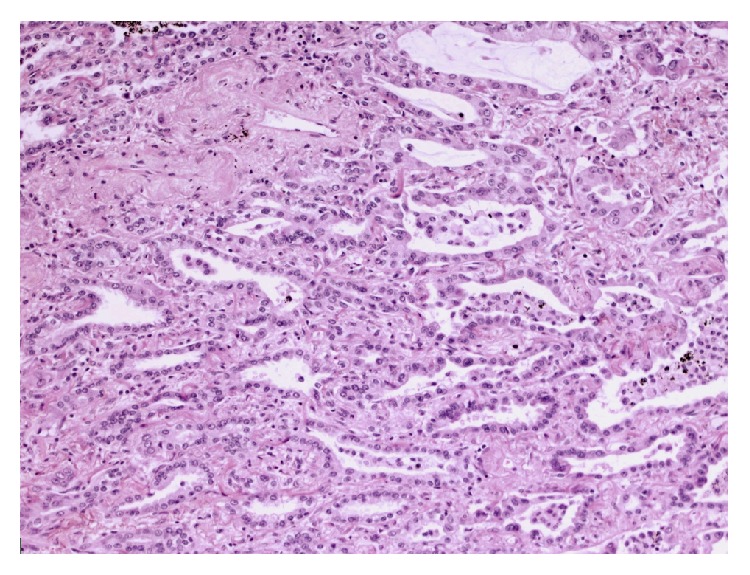
Adenocarcinoma component with acinar growth pattern (H&E ×10 objective).

**Figure 6:**
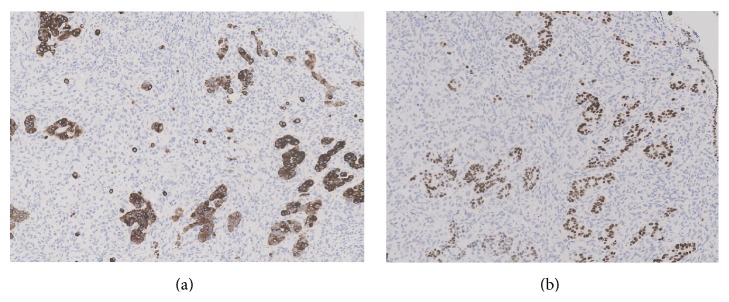
Immunohistochemical stains CK7 and TTF-1 expressed only by the glandular component highlighting the biphasic nature of the tumour (×10 objective).

**Figure 7:**
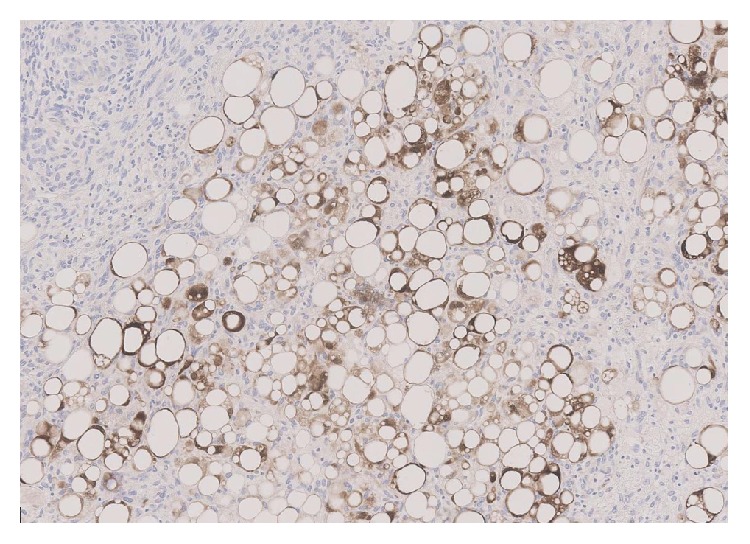
S100 immunohistochemical stain positive in the lipoblastic component (×10 objective).

Subsequent histological examination of the specimen was performed. Sections from lung demonstrated the presence of a rather ill-defined 50 mm mass which microscopically had a heterogeneous appearance (Figure 1). In areas, the tumour was composed of poorly formed epithelial glands which were surrounded by a proliferation of spindle cells (Figure 2). Both the epithelial component and the spindle cell component showed nuclear pleomorphism and a brisk mitotic activity (Figure 3). A heterologous lipoblastic element was observed with the spindle cell component (Figure 4). The sarcomatous component did not express CD99, CD34, CD56, and CDK4. Adjacent and admixed to these areas there were also foci of lung adenocarcinoma with a predominant acinar growth pattern (Figure 5) expressing TTF-1 and CK7 (Figures 6(a) and 6(b)). The glandular component expressed TTF-1 and CK7, while the spindle cell component was negative for all epithelial markers confirming the biphasic nature of the tumour. The lipoblastic element expressed S100 (Figure 7). There was also a focal lepidic pattern of growth seen at the periphery of the specimen.

## 4. Discussion

Pulmonary carcinosarcoma was first described by Kika et al. in 1908 as a poorly differentiated non-small-cell carcinoma containing a component with sarcoma or sarcoma-like features [2].

These tumours tend to present in middle age with the average age of diagnosis around 60 years although patients as young as 31 have been reported [1]. Most patients with pulmonary carcinosarcoma are male and more than 90% have a history of heavy smoking [3].

Preoperative diagnosis of carcinosarcoma is difficult with only a few case reports on the imaging diagnosis of this rare tumour. The tumours are however usually bulky (>5 cm) and have a high tendency to invade adjacent structures such as the pleura, chest wall, diaphragm, or mediastinum. Large low attenuation areas are found on CT that correspond to regions of necrosis and myxoid degeneration. Calcification may be observed within the primary tumour as well as metastases on CT scan, which would likely represent an osteosarcomatous or chondrosarcomatous component to the tumour [4].

Similarly, there is a paucity of data on FDG uptake on PET-CT imaging for these tumours with one report of significantly higher uptake than in other types of lung cancer and another report of an SUV max of 4.1 [1]. Due to the heterogeneity of the tumour, obtaining a tissue diagnosis of carcinosarcomas is difficult as a needle biopsy would often just reflect one element of the tumour.

Surgical resection therefore seems to be the most important part in order to obtain sufficient tissue to make an accurate histological assessment. Clinically, two subtypes have been described: a central endobronchial type and a peripheral invasive type also called parenchymal carcinosarcoma. The peripheral parenchymal type often presents as a large mass. These tumours are often asymptomatic in the early stage, during which time they may involve the adjacent organs or structures such as the mediastinum, pleura, and thoracic wall [5]. The endobronchial lesions are usually slowly growing and locally invasive. The parenchymal tumours tend to be associated with a poorer prognosis as they metastasize early and widely. The carcinomatous component of the tumour is more often squamous cell carcinoma (69%), followed by adenocarcinoma (20%) and large cell carcinoma (11%) [6].

In addition to surgical resection, radiation is well established and effective as the standard therapy in combination with surgery. Even if the sarcoma is resected incompletely and the resection margins show residual tumour on microscopy the rate of local recurrence can be reduced to about 15% by local radiation therapy [6]. Chemotherapy seems to be reserved for cases with remote metastasis [7]. The reported 5-year survival rate is only 21.3% [8]. The tumour has a marked tendency for distant metastasis and has a high rate of local recurrence. Postoperative survival is 9 months on average [9]. However, tumours with endobronchial localization have been reported to have a better prognosis than peripherally located cases as they cause symptoms earlier. The most important predictors of poor prognosis are a size larger than 5 cm [8] and the presence of a rhabdomyosarcoma component which is thought to play a role in the development of haematologic metastases [9].
